# Eco-Friendly Silver Nanoparticles Synthesized from a Soybean By-Product with Nematicidal Efficacy against *Pratylenchus brachyurus*

**DOI:** 10.3390/nano14010101

**Published:** 2023-12-31

**Authors:** Letícia Santana de Oliveira, Leila Lourenço Furtado, Francisco de Assis dos Santos Diniz, Bruno Leonardo Mendes, Thalisson Rosa de Araújo, Luciano Paulino Silva, Thaís Ribeiro Santiago

**Affiliations:** 1 Departamento de Fitopatologia, Universidade de Brasília, Brasília 70910-900, DF, Brazil; leticiasanoli2@gmail.com (L.S.d.O.); leilafurtado24@hotmail.com (L.L.F.); francisco.santos.diniz1996@gmail.com (F.d.A.d.S.D.); bleomendes@gmail.com (B.L.M.); thalissonaraujo76@gmail.com (T.R.d.A.); 2 Laboratório de Nanobiotecnologia (LNANO), Embrapa Recursos Genéticos e Biotecnologia, PBI, Brasília 70770-917, DF, Brazil; luciano.paulino@embrapa.br

**Keywords:** green synthesis, soybean leaf extract, nematotoxic efficacy, root lesion nematode, nematode, nanotechnology

## Abstract

This study explores an eco-friendly approach to synthesizing silver nanoparticles (AgNPs) using soybean leaf extracts, employing a reaction with silver nitrate at 65 °C for 2.5 h. Optimal results were achieved at extract concentrations of 3.12 and 6.25 mg of the leaf mL^−1^, termed 3.12AgNP and 6.25AgNP, respectively. UV-Vis spectrophotometric analysis between 350 and 550 nm exhibited a peak at 410–430 nm, along with a color transition in the suspensions from pale yellow to brown, indicating successful synthesis. Dynamic light scattering (DLS) further delineated the favorable properties of these AgNPs, including nanometric dimensions (73–104 nm), negative charge, and moderate polydispersity, portraying stable and reproducible synthesis reactions. The bioreduction mechanism, possibly expedited by leaf extract constituents such as amino acids, phenolic acids, and polysaccharides, remains to be fully elucidated. Notably, this study underscored the potent nematicidal effectiveness of biosynthesized AgNPs, especially 6.25AgNP, against *Pratylenchus brachyurus*, which is a common plant-parasitic nematode in tropical soybean cultivation regions. In vitro tests illustrated significant nematicidal activity at concentrations above 25 µmol L^−1^, while in vivo experiments displayed a pronounced nematode population diminishment in plant roots, particularly with a 6.25AgNP rhizosphere application at concentrations of 500 µmol L^−1^ or twice at 250 µmol L^−1^, attaining a reproduction factor below 1 without any morphological nematode alterations. This research highlights the potential of 6.25AgNPs derived from soybean leaf extracts in forging sustainable nematicidal solutions, marking a significant stride toward eco-friendly phytonematode management in soybean cultivation. This novel methodology signals a promising avenue in harnessing botanical resources for nematode control and propelling a greener agricultural horizon.

## 1. Introduction

The battle against pests and pathogens is paramount for ensuring global food security, especially for staple crops such as soybean (*Glycine max*). Root lesion nematodes, particularly *Pratylenchus brachyurus* (Godfrey) Filip Schurr-Steekh, pose formidable challenges in this struggle and are known for their devastating impact, as documented by [1,2]. These phytonematodes present a substantial threat to soybean cultivation, causing relevant yield losses due to their wide distribution, extensive host range, and severe, often irreversible, damage [3,4,5].

The presence of *P. brachyurus* is characterized by the emergence of dark, reddish-brown to black lesions on plant roots. Over time, these lesions coalesce, forming extensive necrotic areas that result from the oxidation of root tissues. Additionally, as these nematodes penetrate, they move through root tissues, feed on plant roots, and can establish peculiar interactions with other phytopathogenic microorganisms, ultimately compromising plant health [6,7,8].

The control of root lesion nematode is a highly complex endeavor that requires the simultaneous use of multiple management practices [3]. Presently, the primary control strategies for the *Pratylenchus* group focus on applying chemical compounds, growing nematode-resistant plant varieties, and crop rotation. However, this approach, especially the use of fumigants, is not only inefficient but also poses significant risks for toxicity and is unable to provide complete protection for crops throughout their growth cycle [9,10]. Additionally, the strategies of genetic control and crop rotation each face distinct challenges. The limited availability of soybean-resistant crop varieties and the broad range of host plants susceptible to *P. brachyurus* contribute to the overall ineffectiveness of these methods in substantially reducing nematode populations [11,12].

While a myriad of management strategies have been deployed against *P. brachyurus*, many have fallen short in terms of their long-term sustainability and efficacy [13,14,15]. With traditional methods waning, the scientific community is turning to innovative solutions. Among these, silver nanoparticles (AgNPs), particularly those that are green-synthesized from plant extracts, have emerged as potential game-changers. The prowess of AgNPs derived from various plant extracts in mitigating nematode threats is well-documented, pointing to their promise as potent nematicides.

AgNPs produced through green synthesis methods, particularly using plant leaf extracts, have attracted significant attention due to their effective management of plant-parasitic nematodes [16]. A plethora of recent research has underscored the efficacy of such AgNPs in controlling *Meloidogyne* species through processes such as the inhibition of egg hatching, obstructing the entry of J2 juveniles into roots, and disrupting juvenile development [17,18,19]. Moreover, a study by [20] demonstrated the decline of the *Heterodera sacchari* population and the concurrent enhancement of vegetative growth and yield in rice plants resulting from the application of green-synthesized AgNPs.

The choice of reducing agents in the synthesis of nanoparticles is crucial in determining the physicochemical characteristics and toxic effects of AgNPs. The leaf extracts of plants, which contain compounds like amino acids, flavonoids, phenolic compounds, sugars, and terpenoids, not only reduce silver ions and stabilize the AgNPs but also regulate their growth and aggregation and enhance their nematotoxic activity [21,22,23,24]. For example, AgNPs synthesized using medicinal plant leaf extracts such as *Conyza dioscoridis*, *Melia azedarach*, and *Moringa oleifera* have demonstrated high efficacy against egg hatching and the J2 stage of *M. incognita* [25]. However, secondary metabolites in the *C. dioscoridis* leaf notably enhance the nematicidal effect of AgNPs compared with other treatments. Leaf extracts from a variety of plants, such as *Euphorbia tirucalli*, *Azadirachta indica*, *Curcuma longa*, *Acalypha wilkesiana*, *Senna siamea*, and *Ficus sycomorus*, have been successfully utilized in the biosynthesis of AgNP, demonstrating its effectiveness in reducing phytonematode populations [26,27,28,29,30]. Interestingly, soybean, the very crop threatened by these nematodes, might hold the key to an effective countermeasure. Extracts from soybean leaves, as an often-overlooked by-product, have shown promise for synthesizing potent AgNPs [31,32]. Nevertheless, the potential of these nanoparticles in combating plant parasitic nematodes has yet to be fully explored in research.

Our study delves into this uncharted territory, focusing on *P. brachyurus*, which is a nematode that does not form specialized feeding cells, thus representing a unique challenge. The potential of green-synthesized AgNPs from soybean derivatives is being attempted to harness with a sustainable and cyclical agricultural management approach envisaged. Our overarching goal is to amplify the value of soybean by-products in pest management, offering an innovative, sustainable, and efficient solution.

Given the unexplored potential of AgNPs synthesized from soybean components in controlling phytonematodes, our study sets out to undertake a comprehensive analysis, aiming to (i) assess the effectiveness of AgNPs synthesized through green methods using various concentrations of the soybean leaf extract; (ii) perform the physical and chemical characterization of the synthesized nanoparticles; (iii) evaluate the in vitro and in vivo nematicidal activity of these AgNPs; (iv) investigate the response of biomolecules to the reduction in Ag^+^ to Ag^0^ and the stabilization of the synthesized AgNPs; and (iv) study the mode of action of AgNPs on *P. brachyurus* specimens. By exploring the utilization of soybean-derived AgNPs against root lesion nematodes, this study contributes to the advancement of global food security measures and demonstrates the potential of plant-based technologies in developing safer nematicides.

## 2. Materials and Methods

### 2.1. Soybean Leaf Extract

Healthy soybean plants (cv. Willians) were cultivated in a greenhouse at 25 ± 5 °C at the University of Brasília, Brasília, Federal District, Brazil, for 45 days. The leaves were carefully harvested, washed thoroughly with a 1000× diluted neutral detergent solution, and rinsed with distilled water to remove any residue. The plant material was then air-dried at room temperature and stored in a sealed plastic bag at −20 °C.

To prepare the plant extract, 2 g of the cold leaves were cut and mixed with 20 mL of boiling distilled water for 2 min. The extract was then meticulously filtered through n° 7 filter paper (14 µm) (Qualy Comercial Eireli, Passos, MG, Brazil) using a funnel to remove any solid residues. The resulting filtered aqueous extract had a distinct light green coloration.

### 2.2. Synthesis and Characterization of Nanoparticles

Silver nanoparticles (AgNPs) were synthesized using the soybean leaf extract as the reducing and stabilizing agent. Silver nitrate (AgNO_3_) (Sigma-Aldrich, St. Louis, MO, USA) was selected as the silver salt precursor and dissolved in type I water to a final concentration of 1 mmol L^−1^. The AgNPs reaction syntheses were conducted using varying concentrations of the plant extract at 0.78, 1.56, 3.12, 6.25, 12.5, and 25 mg of the leaf mL^−1^. The final suspensions were denoted as 1.56AgNPs, 3.12AgNP, 6.25AgNP, 12.5AgNP, and 25AgNP, respectively. The stock aqueous extract was diluted to these final concentrations, mixed with the AgNO_3_ solution, and then incubated at 65 °C for 2.5 h. The resulting AgNPs were preserved in a polypropylene tube at 4 °C in the dark for further physical–chemical characterization and the testing of biological activity.

To characterize the nanoparticles, an absorbance curve was generated by collecting measurements from a UV-Vis spectrophotometer (Shimadzu UV-1203, Kyoto, Japan) across a wavelength range of 350 to 550 nm for each reaction synthesis. The peak with the highest intensity indicated the presence of the surface plasmon resonance (SPR) effect. The physical characterization of the AgNPs was then performed using dynamic light scattering (DLS) and electrophoretic mobility techniques on a ZetaSizer Nano ZS (Malvern Instruments, Worcestershire, UK) equipped with a He-Ne laser operating at 633 nm. This methodology facilitated the determination of several parameters, including the hydrodynamic diameter (HD) of the particles, the size distribution of the particle subpopulations evaluated using the polydispersity index (PdI), and the electrophoretic mobility expressed as Zeta potential. The assessments of HD, PdI, and Zeta potential were performed in triplicates, with the measurements automated and the scattering angle set at 173° and 25 °C. Moreover, the reaction syntheses and the measurements of HD, PdI, and the Zeta potential of AgNPs were repeated three times to confirm reproducibility. The data generated were processed using ZetaSizer version 7.11 software developed by Malvern Instruments.

The identification of functional groups in the soybean leaf extract might contribute to the reduction, capping, and stabilization process of AgNPs and was performed using Fourier-transform infrared spectroscopy (FTIR). FTIR spectra were obtained in a potassium bromide (KBr) tablet and in the total attenuated total reflectance (ATR) mode using a Vertex 70 spectrometer (Bruker Corporation, Billerica, MA), outfitted with an ATR configuration specifically tailored for the samples. The samples were deposited onto a diamond crystal and analyzed within a spectral range of 4000 to 350 cm^−1^ and at a resolution of 4 cm^−1^ with a total of 32 scans. The data collected were then acquired and analyzed using OPUS software v7.2 (Bruker Corporation).

### 2.3. P. brachyurus Culture

The culture of the root lesion nematode, *P. brachyurus*, originating from the *Pratylenchus* culture collection of the University of Brasília, was conducted in the Department of Phytopathology, Brasília, Federal District, Brazil. The population was collected from naturally infected soybean plants harvested in a commercial area in the municipality of Luziânia, Goiás, in the Central region of Brazil. The nematode population was propagated in soybean plants growing within a controlled greenhouse environment at 25 °C ± 3 °C.

To confirm the species of the population, nematodes were extracted from the roots of soybean plants cultivated in the greenhouse. The extraction of nematodes was carried out using the methodology described by [33], followed by the molecular, morphological, and morphometric characterization of specimens. For molecular characterization, DNA was extracted from the *Pratylenchus* specimens using the DNeasy Blood and Tissue Kit (Qiagen). A pair of species-specific primers (18S-F and ACM7-R) designed to detect *P*. *brachyurus* was used, and the PCR process was accomplished as described by [34].

For a detailed morphological and morphometric assessment, semi-permanent microscopy slides containing ten adult females were meticulously prepared. Various anatomical structures were studied, including the body length (L), stylet length (ST), stylet bulb diameter (ØSTB), stylet bulb length (STB), tail length (T), esophagus length (ESO), distance from the vulva to anus (VA), largest body diameter (ØL), body diameter at the anus (ØLA), body diameter at the vulva (ØLV), the percentage distance of the vulva from the anterior end (V), and the overlap length of the esophageal glands (EG). Additionally, the De Man indices were also calculated as follows: a (the ratio of body length to the largest body diameter), b (the ratio of body length to esophagus length), c (the ratio of body length to tail length), and c’ (the ratio of tail length to the tail diameter at anus height). The data were statistically analyzed using descriptive statistics, such as the mean and standard deviation. The nematodes were classified according to the identification keys of [3] using a Leica DM2500 optical microscope (Leica, Wetzlar, Germany).

For all bioassay experiments, the inoculum density was calibrated to ~1000 nematodes mL^−1^ using a Peters’ counting chamber with the same optical microscope set to a 40× objective.

### 2.4. Direct Exposure of P. brachyurus to 3.12AgNP and 6.25AgNP

This experiment assessed the inhibitory impact of AgNPs, which were synthesized using different leaf extract concentrations on the mobility of the root lesion nematode, *P. brachyurus,* in an in vitro setting. Two distinct formulations of AgNPs were prepared using 3.12 and 6.25 mg leaf mL^−1^ and were termed 3.12AgNP and 6.25AgNP, respectively. These formulations were chosen based on their physicochemical attributes, which aligned with the requisite criteria for nanomaterials.

The experimental procedure involved exposing 1000 nematodes to six varying concentrations (1, 10, 25, 50, 125, and 250 µmol L^−1^) of both 3.12AgNP and 6.25AgNP formulations independently. The nematode–nanoparticle mixtures were incubated at room temperature for 48 h, after which they were transferred to Baermann funnels for an additional 48 h following the protocol outlined by [35]. Subsequently, live nematodes were harvested from the terminal container after a total incubation period of 96 h and were quantified to evaluate the inhibitory effect under each treatment condition. Sterile water served as the control solution. The experiment was conducted with five experimental units, each represented by one funnel, and was replicated twice at varying time intervals. This resulted in two distinct experimental batches, with a total of ten funnels evaluated under each treatment condition.

The minimum inhibitory concentration (with IC50 and IC90 values, which represent the concentrations required to inhibit 50% and 90% of nematicidal activity, respectively) was ascertained through the analysis of two replicates, followed by logistic regression to fit curves to the data. An analysis of variance (ANOVA) was performed on these values, with treatment means compared using Fisher’s least significant difference test at a significance level of α = 0.05.

### 2.5. AgNP Effects on Soybean Grown and Nematode Population in Soil System

Only the AgNP and concentrations that exhibited the highest nematicidal efficacy in preliminary in vitro mobility studies were selected for in vivo testing. Initially, soybean seeds were sowed in bags filled with a sterilized mixture of soil, sand, and the Bioplant^®^ commercial substrate, mixed in a 1:1:1 ratio. Upon reaching the V2 stage, the rhizosphere of each soybean plant was inoculated with a 1 mL suspension containing 1000 individuals of *P. brachyurus*. Three days after nematode inoculation, a 1 mL dose of the green-synthesized 6.25AgNP was applied to the soil surrounding the rhizosphere. The in vivo nematicidal efficacy of AgNPs was then assessed through two separate experiments, using application timings and concentrations derived from prior in vitro trials.

In the first experiment, a single dose of green-synthesized 6.25AgNP was administered at concentrations of 125 and 250 µmol L^−1^. The second experiment involved a single dose of 6.25AgNP at 500 µmol L^−1^ and two subsequent doses at 250 µmol L^−1^, spaced a month apart. Control treatments consisted of water applications. Throughout the experiment, plants were housed in a greenhouse maintained at a temperature of 25 ± 3 °C for 80 days.

Following 80 days of nanoparticle application, various plant metrics were evaluated, including the plant height, shoot weight, leaf count, root weight, nematodes per gram of root, and the nematode reproduction factor (RF) for each treatment. The RF was calculated based on the contrast between the final population (Pf) and the initial population (Pi), following the methodology proposed by [36]. Nematode data per gram of the root underwent an analysis of variance (ANOVA), with Fisher’s least significant difference calculated to a significance level of *p* = 0.05 for treatment comparisons. Each treatment was composed of eight replicates with a single soybean plant in a 1 L pot. All infection sets were conducted twice at varying intervals.

### 2.6. Transmission Electron Microscopy (TEM) and Scanning Electron Microscopy (SEM)

To determine the average dry size of the most effective AgNPs and investigate their effects on nematode cuticles, a two-pronged microscopy approach was employed as follows: transmission electron microscopy (TEM) for particle analysis and scanning electron microscopy (SEM).

In the first stage, the AgNPs were diluted in water and dispersed onto copper grids for TEM analysis using a JEOL 1011 microscope. The particle diameters were precisely measured using the ImageJ version 1.8.0 software, and the resultant size distribution curve was plotted using R version 3.3.1 software. For the SEM analysis, nematodes were submerged in AgNP suspension for 24 h, rinsed with water, and then fixed in Karnovsky’s solution (2% glutaraldehyde and 2% paraformaldehyde) buffered with 0.05 M of sodium cacodylate at pH 7.2) for 12 h [37]. The nematodes were washed three times with 0.05 M sodium cacodylate and post-fixed with osmium tetroxide for 1 h. Subsequently, they were rinsed with distilled water and dehydrated through a sequential acetone series (50%, 70%, 90%, and twice at 100% concentrations). At each stage of solution disposal, the contents of the tubes were centrifuged at 2500× *g* for 3 min. This process enabled the sedimentation of the nematodes and the complete removal of the supernatant. After dehydration, the nematodes were subjected to critical point drying using a Balzers CPD 030 (BAL-TEC AG, Balzers, Liechtenstein) with carbon dioxide as the transition fluid and mounted on supports adorned with carbon adhesive tape. The final step involved gold sputter coating using a Leica EM SCD 500 (Leica Microsystems, Wetzlar, Germany), after which the samples were ready for SEM analysis on a JEOL JSM-7001F microscope (JEOL, Tokyo, Japan).

## 3. Results

### 3.1. Optical and Physical-Chemical Characterization of Silver Nanoparticles

**Figure 1:**
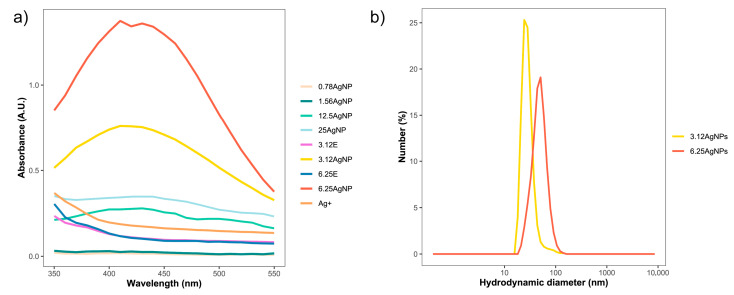
(**a**) Visible absorbance curves of AgNPs and extracts (E) obtained via green synthesis with soy leaf aqueous extract obtained at 65 °C for 2.5 h. (**b**) Hydrodynamic diameter dispersion of AgNPs synthesized using 3.12 (3.12AgNPs) and 6.25 mg mL^−1^ of soybean leaf aqueous extract (6.25AgNPs).

Two rapid methods were used to monitor the successful formation of AgNPs: the transition of the suspension’s color from yellow to browni upon mixing the aqueous leaf extract with the AgNO_3_ solution and the emergence of peaks at a wavelength of approximately 425 nm in spectrophotometric analyses. Optical properties revealed that free silver ions (Ag^+^), extracts (E), and the lowest concentration of the extract (1.56 mg of leaf mL^−1^) mixed with AgNO_3_ did not induce any color changes in the solution. On the other hand, all other reaction media containing various concentrations of the leaf soybean extract and AgNO_3_ exhibited a color transition from yellow to brown (Figure S1), suggesting the formation of nanoparticles. Absorbance spectra were measured in the visible region of 350–550 nm using UV-Vis spectroscopy (Figure 1a). Absorbance peaks in the range of 410–430 nm were exclusively detected in the 3.12AgNP and 6.25AgNP suspensions, confirming the formation of AgNPs. Notably, the absorbance of these nanoparticles varied, with higher values associated with higher leaf extract concentrations in the reaction. A reduced absorbance peak was observed in the 12.5AgNP and 25AgNP. No such peaks were identified in the other synthesis reactions, implying the lack of an SPR effect.

The physical characteristics of the nanoparticles synthesized were analyzed using DLS and electrophoretic mobility. The 12.5AgNP and 25AgNP exhibited a high HD (>450 nm) and expressive precipitation; therefore, the possible nematicide activities of these nanoparticles were not evaluated. Otherwise, the 3.12AgNP and 6.25AgNP had an HD of 104.4 ± 2.4 nm and 73.5 ± 1.5 nm, respectively (Table 1). They exhibited a homogeneous size distribution, forming a uniformly singular population (Figure 1b). The concentration of the extract appeared to influence the nanoparticle size, but the potential values of PdI and Zeta were similar across both concentrations. The extract concentrations tested resulted in a relatively low PdI ranging from 0.23 ± 0.01 to 0.22 ± 0.02 (Table 1). The AgNPs exhibited a negative Zeta potential ranging from −25.1 ± 0.5 to −23.3 ± 0.2 mV (Table 1). These values indicated that the AgNPs possess moderate colloidal stability due to sufficient mutual electrostatic repulsion to maintain suspension stability. No differences in size, PdI, and Zeta potential were observed among the three reaction syntheses in both concentrations of the leaf extract (Table 1).

**Figure 2:**
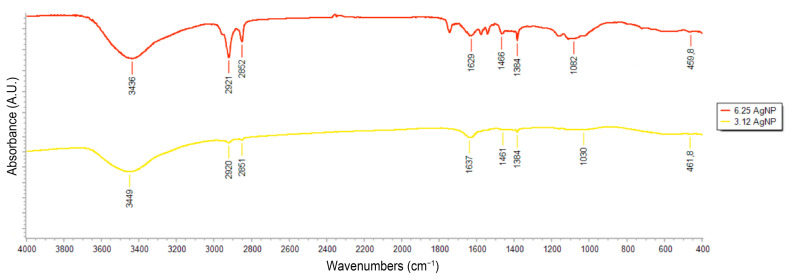
Fourier-transform infrared spectroscopy (FTIR) spectra of AgNPs synthesized using 3.12 (3.12AgNPs; yellow line) and 6.25 mg mL^−1^ soybean leaf aqueous extract (6.25AgNPs; red line).

Functional groups in biomolecules from the soybean leaf extract, potentially involved in the reduction in Ag^+^ and synthesis of 3.12AgNP and 6.25AgNP, were investigated using FTIR measurements. The absorbance bands identified in the spectra of the soybean extract were the same in both syntheses and located at 3449, 2920, 2851, 1637, 1461, 1384, 1032, and 461.8 cm^−1^ (Figure 2). Different concentrations of the soybean leaf extract resulted in the detection of the same elements in the FTIR profile, with extracts containing 6.25 mg mL^−1^ exhibiting a higher intensity for all peaks. Multiple peaks were observed in the analysis, which are described according to [38] as follows: the peak at 3449 cm^−1^ corresponds to the stretching vibration of hydroxyl (OH) groups, commonly found in alcohols and phenols; the peaks at 2920 cm^−1^ and 2851 cm^−1^ are indicative of the vibration of aliphatic C-H bonds, typical in alkanes and alkyl groups; the peak at 1637 cm^−1^ is associated with an amide absorption band, suggesting the presence of peptide bonds in proteins; the peak at 1461 cm^−1^ is related to methyl groups; the peak at 1384 cm^−1^ is associated with various types of bonds, including C-H bonds in alkenes or ketones; the peak at 1032 cm^−1^ indicates a C-O band, which is characteristic of esters, alcohols, and other functional groups; lastly, the peak at 461.8 cm^−1^ is related to vibrations in metal bonds or bending modes in complex molecules.

### 3.2. In Vitro and In Vivo Nematicidal Activity of AgNPs

The *P. brachyurus* specimens were identified using PCR amplification with the species-specific primers 18S-F/ACM7-R and morphological analysis. The PCR process yielded a single fragment approximately 270 bp in length, confirming the identification of *P. brachyurus* [34]. All morphological and morphometric measurements also indicated the species *P. brachyurus* (Table S1). Females exhibited body lengths and widths that ranged from 534.67 to 577.83 µm and 22.44 to 23.48 µm, respectively. The cephalic region is slightly offset from the main body, with the oral aperture featuring two distinct sclerotized lip annuli. The stylet is robust, with a length varying between 19.23 and 20.63 µm. It is distinguished by its tulip-shaped nodules, which maintain a relatively uniform width ranging from 4.35 to 4.68 µm. The medium bulb is robust and oval-shaped.

The length of the esophagus ranges from 81.47 to 87.09 µm. The esophageal glands, which overlap the intestine on the ventral and lateral sides, measure about 52.62 ± 1.14 µm. The vulva is located 452 to 482 µm from the anterior end, accounting for approximately 82.62% to 86.50% of the total body length. The reproductive system is monodelphic-prodelphic, with a non-functional spermatheca. The tail shape varies from club-shaped to truncated to conical, with a length of 28.56 to 31.76 µm. Males were not found (Figure S2).

**Figure 3:**
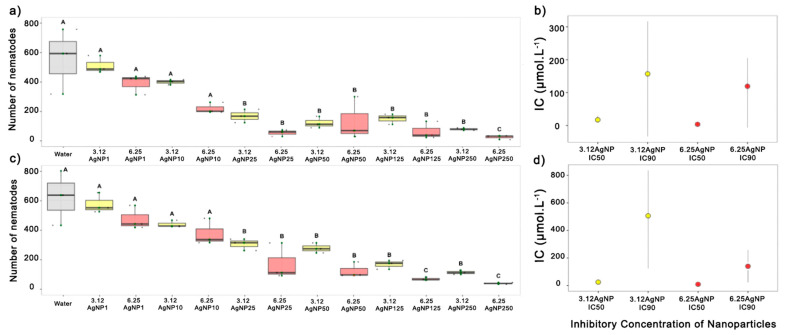
Evaluation of the impact on the mobility of *Pratylenchus brachyurus* specimens exposed to six different concentrations (1, 10, 25, 50, 125, and 250 µmol mL^−1^) of the AgNPs. The nanoparticles were synthesized with 3.12 (3.12AgNP) and 6.25 mg mL^−1^ of the aqueous soy leaf extract (6.25AgNP). The study was conducted at room temperature over a period of 48 h in two sequential in vitro experiments, identified as the first (**a**) and second (**c**) trials. IC50 and IC90 values and the confidence interval (bar) of AgNPs of treatment against *P. brachyurus* in the first (**b**) and second (**d**) in vitro experiments. 3.12AgNP and 6.25AgNPs are represented by the yellow and red color bars, respectively. The application of water was used as a control and is represented by a gray color bar. The mean number of nematodes/gram of the soybean root was calculated using the mean of eight replicates. The same letters indicate no significant difference according to the LSD test.

After identifying the nematodes, the effects of different concentrations of AgNPs on their mobility and mortality were tested both in vitro and in vivo, respectively. After incubating the *P. brachyurus* specimens with 3.12AgNP and 6.25AgNP for 48 h, the nanoparticles significantly inhibited the nematode mobility at a concentration of 25 µmol L^−1^ or higher compared to the control (*p* < 0.05) (Figure 3a–d). In one experiment, 6.25AgNP was more effective at high concentrations (125 and 250 µmol L^−1^) (Figure 3c) (*p* < 0.05). Although both AgNPs had nematotoxic effects, a sigmoidal logistic curve was constructed, and IC50 and IC90 were calculated to determine which nanoparticle was most toxic to the phytonematode.

A sigmoidal logistic curve was fitted to the nematicidal effect progress with an R^2^ value above 0.90, and the SE values were low, indicating a good model fit to the data in both experiments. Based on the logistic curves, IC50 and IC90 values were calculated, and the lowest values were found for 6.25AgNPs, with IC50 values ranging from 3.66 (0.85–8.19) to 9.6 (0.68–12.9) µmol L^−1^, and IC90 of 119.29 (6.93–205.52) to 140.09 (22.35–212.53) µmol L^−1^. This is compared to an IC50 range of 17.21 (8.65–28.77) to 25.18 (13.65–36.71) µmol L^−1^ and an IC90 range of 157.02 (33.66–316.70) to 506.45 (123.33–836.26) µmol L^−1^ for 3.12AgNPs (Table 2; Figure 3b,d). Because 6.25AgNPs are the most effective in inhibiting the mobility of *P. brachyurus* in vitro, only this AgNP at the concentrations of 125 and 250 µmol L^−1^ was tested in vivo to investigate its potential to interfere with multiplication and/or lead to nematode death.

**Figure 4:**
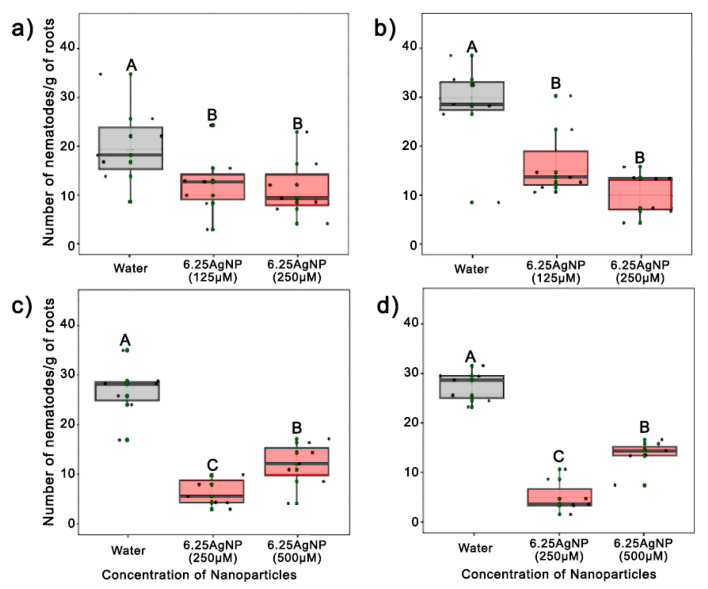
(**a**,**b**) The impact of a single application of nanoparticles synthesized with 6.25 mg mL^−1^ of the aqueous soy leaf extract (6.25AgNP) at concentrations of 125 µmol L^−1^ and 250 µmol L^−1^, on the proliferation of *Pratylenchus brachyurus* specimens per gram of the treated soybean root. (**c**,**d**) The impact of a single application of 6.25AgNP at a concentration of 500 µmol L^−1^ and two applications of 250 µmol L^−1^ spaced one month apart on the proliferation of *Pratylenchus brachyurus* specimens per gram of the treated soybean root. The application of water was used as a control. The number of nematodes was scored after 80 days of nematode inoculation. The application of 6.25AgNPs and water are represented by the red and gray color bars, respectively. The mean number of nematodes/gram of the soybean root was calculated using the mean of eight replicates. The same letters indicate no significant difference according to the LSD test.

In the first set of in vivo experiments, the application of 6.25AgNP at a concentration of 125 and 250 µmol L^−1^ differed from the control in the number of nematodes per gram of the root by up to 62%, with no phytotoxicity observed in either experiment (*p* < 0.05) (Figure 4a,b). Otherwise, the FRs were greater than one, indicating that the nematode population multiplied in the root when 6.25AgNP was applied once at a concentration of 125 and 250 µmol L^−1^. To try to inhibit the phytonematode multiplication, the experiment was repeated by applying 6.25AgNP at a concentration of 250 µmol L^−1^ three days after nematode inoculation and again after 30 days. The application of nanoparticles once at a concentration of 500 µmol L^−1^ and three days after inoculation was also evaluated. Increasing the number of nanoparticle applications and using a higher concentration enhanced the in vivo nematicidal activity against *P. brachyurus*, resulting in an FR < 1 in both treatments. Furthermore, two applications of 6.25AgNP at a concentration of 250 µmol L^−1^ were more effective than one application of AgNPs at a concentration of 500 µmol L^−1^ (*p* < 0.05). No statistical differences were observed in the plant height, shoot weight, leaf count, or root weight with the application of different concentrations of nanoparticles in the soil.

### 3.3. 6.25AgNP Dry Size and Mode of Action on Phytonematodes

**Figure 5:**
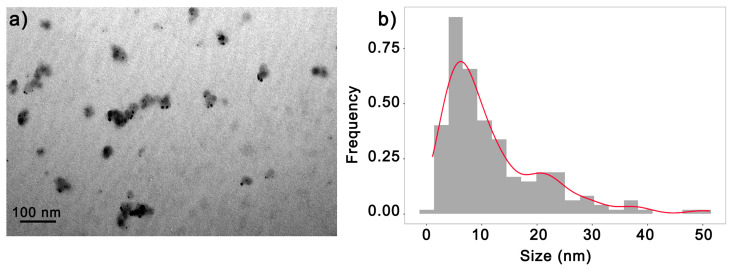
(**a**) Images obtained via the transmission electron microscopy (TEM) of AgNPs synthesized using 6.25 mg mL^−1^ of the soybean leaf aqueous extract (6.25AgNPs) and (**b**) size distribution of particles for 6.25AgNPs in dry condition.

To better understand the dry diameter size and shape of the synthesized 6.25AgNP, as well as their improved physicochemical properties and nematicidal activity, TEM analysis was employed. This analysis revealed the presence of spherical AgNPs with an average size of 11.89 ± 9.28 nm. Some degree of agglomeration was observed, accompanied by moderate-sized variations (Figure 5a,b).

**Figure 6:**
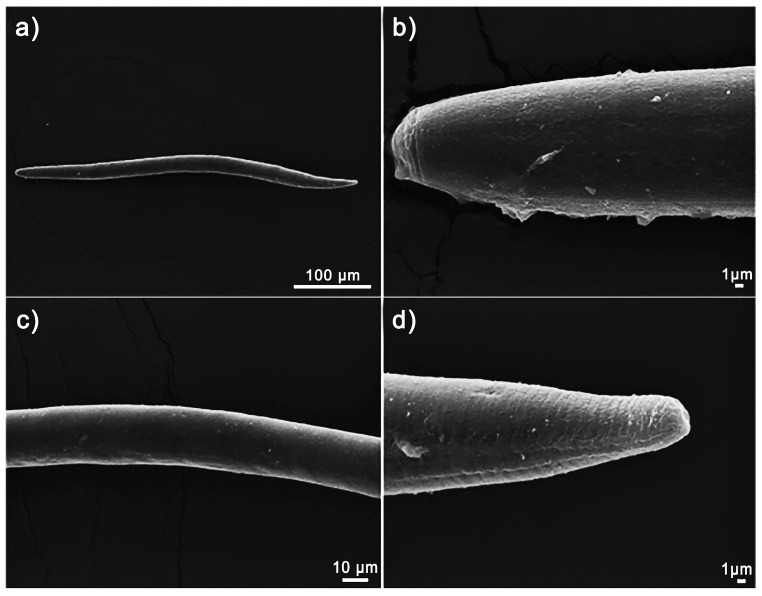
Scanning electron microscopy (SEM) images were taken after exposing the *Pratylenchus brachyurus* specimens in a 250 μmol L^−1^ solution of AgNPs (synthesized using 6.25 mg mL^−1^ of soy leaf aqueous extract, termed 6.25AgNPs) for a period of 48 h. The images depict various parts of the *P. brachyurus* adult: (**a**) the body, (**b**) the labial region, (**c**) the median region, and (**d**) the tail terminus.

The examination of SEM images suggests that the 6.25AgNPs initially adhere to the nematode cuticle. However, no discernible alterations in the cuticle were detected during the exposure period to AgNPs (Figure 6a–d).

## 4. Discussion

Modern agricultural research aims to promote sustainable practices that reduce reliance on external inputs and that foster biodiversity. This involves employing non-polluting techniques and repurposing all by-products. Our study delves into this concept, demonstrating the use of lesser-utilized soybean by-products as reliable agents to reduce silver ions, generating AgNPs. These nanoparticles provide an eco-friendly solution to control the destructive phytonematode *P. brachyurus*, which is an important threat to tropical soybean crops. Using soybean leaf extract, silver ions were efficiently converted into spherical nanoparticles characterized by uniform distribution, low polydispersity, and hydrodynamic diameters ranging from 73 to 104 nm.

While previous research has explored the green synthesis of these nanoparticles through the soybean leaf extract [31,32], this study investigated the reproducibility of the reaction synthesis at different intervals. It also evaluated how fluctuating extract concentrations affect the physical and biological attributes of the AgNPs. The formation of AgNPs using leaf concentrations of 3.12 and 6.25 mg mL was confirmed, evidenced by color changes and UV-Vis absorption peaks (410–430 nm), demonstrating the SPR effect. Additionally, an increase in absorbance as the extract concentration in the reactions increased was observed, as shown in Figure 1a. This enhanced absorbance, as a manifestation of the SPR effect, could indicate alterations in the AgNPs’ size, shape, composition, reduction yield, surface characteristics, or potential shifts in their biological activity [39].

Our findings suggest that doubling the concentration of the leaf extract could lead to a decrease in AgNPs’ size while enhancing its effectiveness against *P. brachyurus*. However, concentrations lower than 3.12 mg and higher than 6.25 mg of the extract per mL inhibited a reduction in silver ions, thereby preventing AgNP formation. This was evidenced by either minimal absorbance peaks below 400 nm or the absence of the formation of nanometric particles sized less than 100 nm. The presence and/or concentration of specific constituents within the soybean extract could either inhibit or promote AgNP formation, as well as affect their dimensions and form [40,41]. Achieving reproducible physicochemical properties of AgNPs presents a significant challenge, given that minor variations in plant growth conditions can alter the properties of secondary metabolites responsible for the reduction in silver ions [42,43,44,45]. A noteworthy point from our study is the observed absence of reproductive anomalies, which might be attributed to the uniform handling of soybean leaves. These leaves were collected simultaneously and stored securely until utilized for the reaction syntheses, ensuring consistency in the material used.

Various studies have highlighted a range of biomolecules that are pivotal in the formation and properties of nanoparticles, including polysaccharides, polyphenols, and alcohols [46,47,48]. Building on this knowledge, the specific functional groups in soybean leaves that facilitate the reduction in Ag⁺ ions to Ag^0^ were further investigated. FTIR analysis was employed to provide useful insights into the mechanisms underlying the synthesis of AgNPs mediated by soybean leaf extracts. Our results showcase the complex and multifunctional nature of compounds derived from soybean aqueous extracts, demonstrating a variety of biomolecule functional groups that can facilitate the reduction in metal ions. The presence of signals corresponding to alcohols and phenols, along with amide absorption bands, indicates the likely involvement of phenolic acids and polysaccharides in the reduction process. This is consistent with earlier research that emphasized the expressive silver ion binding capacities of amino acids and polysaccharides present in soybean leaves [49]. Other studies have proposed that agents such as flavonoids and isoflavones [50,51], as well as soluble soybean polysaccharides [52,53,54], could also act as potential reducing agents in this process.

Although our synthesized AgNPs demonstrated varied toxicity towards *P. brachyurus,* they did not have any negative effects on soybean plants. In a broader context, different studies suggest that exposure for up to 3 days at 100–800 µmol L^−1^ is needed to achieve noticeable reductions in phytonematode mobility or mortality [55,56,57]. In our experiments, it was observed that a 48 h exposure with AgNPs was required to neutralize over 50% of the nematode population’s mobility (IC50) at concentrations ranging from 3.66 to 25.18 µmol L^−1^. Our findings align with [58], who reported similar effective concentrations for marine algae-derived AgNPs against tomato-infecting nematodes. Notably, even at identical concentrations, there were discernible differences in the effectiveness between the 3.12AgNP and 6.25AgNP treatments. The latter AgNPs demonstrated superior results. This can be attributed to the consensus that smaller nanoparticles, due to their increased surface area, display enhanced reactivity and efficacy [59,60]. Moreover, in vitro experiments revealed a greater reduction in nematode mobility when exposed to AgNP concentrations of 125 and 250 µmol L^−1^, especially to 6.25AgNP.

These promising in vitro results were not replicated in in vivo trials with *P. brachyurus*. In the soybean rhizospheres, nematode proliferation at a concentration of 125 and 250 µmol L^−1^ reduced the number of nematodes in the root compared to the control group. However, surprisingly, both concentrations resulted in a reproduction factor (RF) greater than one, indicating nematode reproduction even after the application of AgNPs. The contrast between in vitro and in vivo findings highlights the complex interplay of AgNP behavior within the soil environment. This complexity can be attributed to various factors, including the absorption of AgNPs by plant roots and their attachment to soil particles, resulting in dynamic nanoparticle distributions in the vicinity of the roots [61] and their interactions with the multifaceted rhizosphere microbial community [62]. Such behavior, highlighted in recent studies like that of [63], points to the intricate challenge of predicting nanoparticle effects in natural settings and underscores the need for extensive research to unravel the underlying mechanisms at play.

Another contributing factor could be that AgNPs show diminished effectiveness, possibly because of the nematodes’ ability to evade, especially in mobile species like the *Pratylenchus* species, or the lack of correlation between the dose to inhibit mobility and mortality. Importantly, the discrepancy in outcomes was lessened when either a single high-dosage application of 500 µmol L^−1^ was used and mainly when 250 µmol L^−1^ was administered repeatedly in a second assay. Repeated exposure to the AgNPs may cause a cumulative or enhanced effect on the nematode population, disrupting their development or survival over time. This paves the way for further exploration into optimizing dosage strategies to harness the nematicidal potential of AgNPs.

An in-depth analysis using SEM depicted the accumulation of AgNPs on the nematode cuticle, albeit without apparent immediate alterations to the cuticle, highlighting the need for further research to understand the prolonged influence of AgNPs on nematode physiology and behavior. Previous studies have suggested that AgNPs affect phytonematodes through complex and diverse cellular mechanisms [64]. AgNPs have the potential to induce adverse effects, including germ cell death, reproductive problems, reduced life expectancy, and genetic damage across generations, as nematodes internalize these particles [65,66,67,68].

In accordance with environmental regulations, it is worth noting that regulatory agencies, including the Brazilian Ministry of Health, have established a permissible level for elemental silver in drinking water at 50 µg mL^−1^ [69]. In our study, the applied silver concentrations consistently remained below this threshold, measuring at 0.084 µg mL^−1^. Our findings underscore the relevance of sustainable nematode management in soybean cultivation. Green-synthesized AgNPs exhibit remarkable nematicidal action against *P. brachyurus*, positioning them as a viable alternative to traditional chemical-based solutions. Their eco-friendliness further accentuates their appeal, alleviating environmental pollution concerns tied to conventional methods.

The potential of AgNPs as a nematode control strategy necessitates further exploration. Elucidating the long-term effects on nematode physiology and their reproductive, developmental, and behavioral aspects is vital. Understanding AgNPs’ nematicidal action and the absorbance of AgNPs in soil can refine their field application and dosage. Assessing their impact on non-target organisms and soil health can ensure their safe and sustainable application in tropical soybean cultivation regions.

In essence, our study unveils the promise of green-synthesized AgNPs against *P. brachyurus* in soybean agriculture. They emerge as an environmentally conscious and sustainable tool against nematode infestations. This research contributes substantially to the broader understanding of AgNPs’ biological capabilities and practical applications, marking a pivotal move towards greener nematode control solutions in the agricultural realm.

## Figures and Tables

**Table 1:** Physicochemical characteristic of the AgNPs synthesized with 3.12 (3.12AgNP) and 6.25 (6.25AgNP) mg mL^−1^ of the soy leaf aqueous extract obtained via dynamic light scattering to acquire the hydrodynamic diameter, polydispersity index (PdI), and Zeta potential synthesized three times.

	First Synthesis			Second Synthesis			Third Synthesis		
Nanoparticles	Size (d·nm)	PdI	Zeta Potential (mV)	Size (d·nm)	PdI	Zeta Potential (mV)	Size (d·nm)	PdI	Zeta Potential (mV)
Ag^+^	64.7 ± 4.3	0.57 ± 0.04	−19.1 ± 1.3	-	-	-	-	-	-
3.12AgNP	104.3 ± 2.4	0.23 ± 0.01	−25.1 ± 0.5	101.1 ± 3.4	0.19 ± 0.01	−28.1 ± 0.5	113.3 ± 1.7	0.22 ± 0.02	−20.2 ± 1.4
6.25AgNP	73.5 ± 1.5	0.22 ± 0.02	−23.3 ± 0.2	70.2 ± 2.2	0.20 ± 0.03	−23.3 ± 0.2	79.1 ± 1.8	0.19 ± 0.05	−19.7 ± 2.1

**Table 2:** IC50 and IC90 values and the confidence interval (parentheses) of AgNPs for treatment against *Pratylenchus brachyurus* in first and second in vitro experiments.

Nanoparticles	Experiment_1	Experiment_2
3.12AgNP_IC50	17.21 (8.65–28.77)	25.18 (13.65–36.71)
6.25AgNP_IC50	3.66 (0.85–8.19)	9.6 (0.68–12.9)
3.12AgNP_IC90	157.02 (33.66–316.7)	506.45 (123.33–836.26)
6.25AgNP_IC90	119.29 (6.93–205.52)	140.09 (22.35–212.53)

## Data Availability

Data are contained within the article.

## Supplementary Materials

The following supporting information can be downloaded at: https://www.mdpi.com/article/10.3390/nano14010101/s1, Figure S1: Tubes displaying a shift in color from yellow to brown at different concentrations, indicating success in the synthesis of silver nanoparticles. Figure S2: (**a**) Photomicrograph of *Pratylenchus brachyurus* females showcasing the sausage-shaped body with the vulva position indicated by an arrow. (**b**) The cephalic region slightly offset from the main body, with the oral aperture featuring two distinct sclerotized lip annuli, a robust stylet, and tulip-shaped nodules. (**c**) Esophageal glands overlap the intestine ventrally and laterally with a robust medium bulb. (**d**) The tail’s morphology is characterized as club-shaped, truncated, and conical.; Table S1: Morphological and morphometric characterization of *Pratylenchus brachyurus:* overall body length (L), tail size (T), esophagus length (ESO), stylet length (ST), stylet bulb length (STB), diameter of stylet bulb (ØSTB), distance of vulva to anus (VA), largest body diameter (ØL), body diameter at anus height (ØLA), body diameter at vulva height (ØLV), % vulva from the anterior end (V; V%), length of overlap of esophageal glands (EG), a (relationship L/ØL), b (relationship L/ESO), c (relationship L/T) and c’ (relationship T/ØLA). Data are means of 10 adult female specimens.
