# Supplementary material for: Eco-Friendly Silver Nanoparticles Synthesized from a Soybean By-Product with Nematicidal Efficacy against Pratylenchus brachyurus
Source: Nanomaterials (Basel). 2023 Dec 31;14(1):101. doi: 10.3390/nano14010101 (PMC10780907; doi:10.3390/nano14010101)
Supplement: Supplementary file 1 [file nanomaterials-14-00101-s001.zip › nanomaterials-2734297-supplementary.pdf]

SUPPLEMENTARY MATERIAL

Eco-Friendly Silver Nanoparticles Synthesized from a Soybean By-Product with Nematicidal Efficacy against *Pratylenchus brachyurus*

Letícia Santana de Oliveira [1], Leila Lourenço Furtado [1], Francisco de Assis dos Santos Diniz [1], Bruno Leonardo Mendes [1], Thalisson Rosa de Araújo [1], Luciano Paulino Silva [2] and Thaís Ribeiro Santiago [1,*]

[1] Departamento de Fitopatologia, Universidade de Brasília, Brasília 70910-900, DF, Brazil; leticiasanoli2@gmail.com (L.S.d.O.); leilafurtado24@hotmail.com (L.L.F.); francisco.santos.diniz1996@gmail.com (F.d.A.d.S.D.); bleomendes@gmail.com (B.L.M.); thalissonaraujo76@gmail.com (T.R.d.A.)

[2] Laboratório de Nanobiotecnologia (LNANO), Embrapa Recursos Genéticos e Biotecnologia, PBI,
Brasília 70770-917, DF, Brazil; luciano.paulino@embrapa.br

* Correspondence: thais.santiago@unb.br

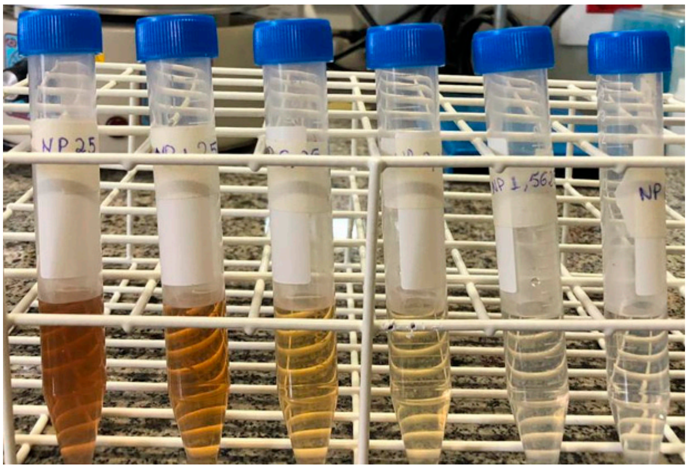

Figure S1: Tubes displaying a shift in color from yellow to brown at different concentrations, indicating success in the synthesis of silver nanoparticles.

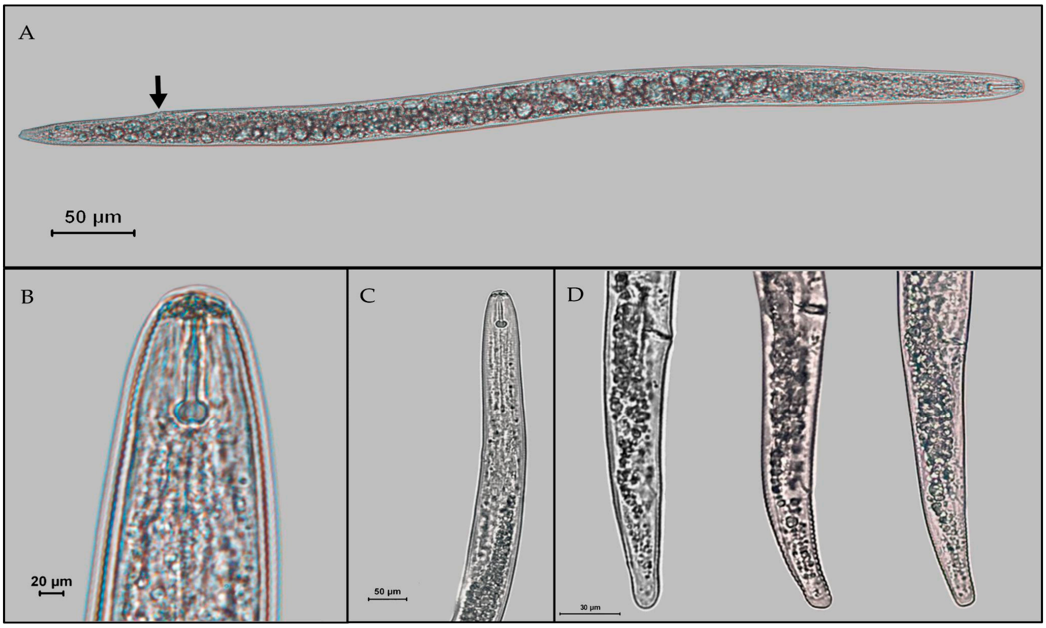

Figure S2: (**a**) Photomicrograph of *Pratylenchus brachyurus* females showcasing the sausage-shaped body with the vulva position indicated by an arrow. (**b**) The cephalic region slightly offset from the main body with the oral aperture featuring two distinct sclerotized lip annuli, a robust stylet, and tulip-shaped nodules. (**c**) Esophageal glands overlap the intestine ventrally and laterally with a robust medium bulb. (**d**) The tail's morphology is characterized as club-shaped, truncated, and conical.

Table S1. Morphological and morphometric characterization of *Pratylenchus brachyurus*: overall body length (L), tail size (T), esophagus length (ESO), stylet length (ST), stylet bulb length (STB), diameter of stylet bulb (ØSTB), distance of vulva to anus (VA), largest body diameter (ØL), body diameter at anus height (ØLA), body diameter at vulva height (ØLV), % vulva from anterior end (V; V%), length of overlap of esophageal glands (EG), a (relationship L/ØL), b (relationship L/ESO), c (relationship L/T) and c′ (relationship T/ØLA). Data are means of 10 adult female’s specimens.

Parameters: mean - standard deviation (SD)

	**L (mm)**	**T (µm)**	**ESO (µm)**	**ST (µm)**	**STB (µm)**	**ØSTB (µm)**	**VA (µm)**	**ØL (µm)**	**ØLA (µm)**	**ØLV (µm)**	**V (mm)**	**V%**	**EG (µm)**	**a**	**b**	**c**	**c'**
SPECIMEN 1	0,534	31,12	88,76	19,87	3,48	4,12	54,1	23,7	15,12	21,34	0,46	86	53,2	22,54	6,02	17,17	2,06
SPECIMEN 2	0,545	28,22	83,23	20,12	3,22	4,23	49,14	22,8	16,01	22,8	0,462	85	50,19	23,91	6,55	19,32	1,76
SPECIMEN 3	0,524	31,1	90,01	19,76	4,1	4,55	53,99	22,32	15,99	21,54	0,463	88	52,32	23,47	5,82	16,85	1,94
SPECIMEN 4	0,546	30,55	83,45	18,99	3,98	4,01	53,76	22,78	15,76	21,65	0,467	86	51,46	23,95	6,54	17,86	1,94
SPECIMEN 5	0,591	27,88	81,19	19,42	3,78	4,19	48,74	23	15,7	20,67	0,486	82	53,87	25,71	7,28	21,21	1,78
SPECIMEN 6	0,563	29,11	82,87	20,87	4,21	4,29	50,11	22,97	15,43	22,13	0,472	84	53,76	24,53	6,80	19,35	1,89
SPECIMEN 7	0,560	31,29	83,91	19,01	3,91	5,1	47,89	23,14	15,14	21,49	0,485	87	52,91	24,20	6,67	17,90	2,07
SPECIMEN 8	0,543	29,34	83,75	20,49	3,68	4,03	51,02	22,29	15,81	22,99	0,452	83	53,61	24,37	6,49	18,51	1,86
SPECIMEN 9	0,582	33,18	82,71	19,74	3,99	4,33	45,66	23,89	16,22	21,29	0,482	83	52,77	24,36	7,04	17,54	2,05
SPECIMEN 10	0,574	29,77	82,89	20,99	4,54	4,61	48,91	22,71	15,83	21,21	0,472	82	52,13	25,26	6,92	19,27	1,88
**MEAN**	**0,556**	**30,16**	**84,28**	**19,93**	**3,89**	**4,35**	**50,33**	**22,96**	**15,70**	**21,71**	**0,47**	**84,57**	**52,62**	**24,23**	**6,61**	**18,50**	**1,92**
**SD**	**± 0,021**	**± 1,6**	**± 2,8**	**± 0,7**	**± 0,37**	**± 0,33**	**± 2,86**	**± 0,51**	**± 0,36**	**± 0,72**	**± 0,011**	**± 1,95**	**± 1,14**	**± 0,88**	**± 0,44**	**± 1,31**	**± 0,11**
